# Combination treatment of cancer cells with pan-Akt and pan-mTOR inhibitors: effects on cell cycle distribution, p-Akt expression level and radiolabelled-choline incorporation

**DOI:** 10.1007/s10637-018-0642-5

**Published:** 2018-07-28

**Authors:** Su Myat Phyu, Tim A. D. Smith

**Affiliations:** 1 School of Medicine, Medical Sciences and Nutrition, University of Aberdeen, Aberdeen, AB25 2ZD UK; 2 Biomedical Physics Building, University of Aberdeen, Foresterhill, Aberdeen, AB25 2ZD UK

**Keywords:** Breast cancer, Radiolabelled choline, Cell cycle, Akt, Combination index

## Abstract

Signal transduction pathways, which regulate cell growth and survival, are up-regulated in many cancers and there is considerable interest in their pharmaceutical modulation for cancer treatment. However inhibitors of single pathway components induce feedback mechanisms that overcome the growth moderating effect of the inhibitor. Combination treatments have been proposed to provide a more complete pathway inhibition. Here the effect of dual treatment of cancer cells with a pan-Akt and a pan-mTOR inhibitor was explored. Breast (SKBr3 and MDA-MB-468) and colorectal (HCT8) cancer cells were treated with the pan-Akt inhibitor MK2206 and pan-mTOR inhibitor AZD8055. Cytotoxic effect of the two drugs were determined using the MTT assay and the Combination Index and isobolomic analysis used to determine the nature of the interaction of the two drugs. Flow cytometry and western blot were employed to demonstrate drug effects on cell cycle distribution and phosph-Akt^ser473^ expression. Radiolabelled ([methyl-^3^H]) Choline uptake was measured in control and drug-treated cells to determine the modulatory effects of the drugs on choline incorporation. The two drugs acted synergistically to inhibit the growth rate of each cancer cell line. Flow cytometry demonstrated G0/G1 blockade with MK2206 and AZD8055 which was greater when cells were treated with both drugs. The incorporation of [methyl-^3^H] choline was found be decreased to a greater extent in cells treated with both drugs compared with cells treated with either drug alone. *Conclusions* Pan-mTOR and pan-Akt inhibition may be highly effective in cancer treatment and measuring changes in choline uptake could be useful in detecting efficacious drug combinations.

## Introduction

Cancer growth and development are frequently associated with upregulation of intracellular signalling pathways, including the PI3K/Akt/mTOR pathway, which drives cellular proliferation resulting in the uncontrolled cell growth that defines cancer [1]. The increased levels of activation of PI3K/Akt/mTOR can be the result [2] of activating mutations of genes coding for PI3K or Akt (also known as PKB (protein kinase B)), kinases that stimulate the pathway, or inactivating mutations in the gene for the inhibitory phosphatase, PTEN (phosphatase and tensin homolog).

In view of the central role of PI3K/Akt/mTOR in cancer cell proliferation in many tumour types there is much interest in oncology in the use of inhibitors targeting components of this pathway [3]. Although the use of such inhibitors has been shown to decrease cancer cell proliferation and survival, their efficacy as single-agents is poor [4] due to activation of feedback mechanisms [5, 6] so attenuating the clinical benefit from the drug. Consequently their use in combination treatments has been recommended [7] and it has been shown that drug combinations, including the dual use of Akt and mTOR inhibitors, are more efficacious than single drug regimens as they bring about a more complete pathway inhibition [8]. However drugs targeting the PI3K/Akt/mTOR pathway are associated with unpleasant and potentially dangerous side effects including nausea, diarrhoea and hyperglycaemia [9] which may be enhanced in drug combinations [8].

Therefore, early detection of the clinical benefit of multi-drug regimens is an important aspect of their utilisation. Positron emission tomography (PET) is a highly sensitive medical imaging technique that facilitates non-invasive measurement of metabolism. The most commonly used PET tracer is FDG (2-[^18^F] Fluor-2-deoxy-D-glucose) and many studies have demonstrated that changes in its tumour incorporation accompanies treatment response. However this is not always the case for drugs targeting the PI3K/Akt/mTOR pathway [10]. Choline uptake has been shown to be associated with proliferation rate [11] and [^11^C] choline-PET has been applied to response detection to treatments that target receptors upstream of signalling pathways [12]. Here we have demonstrated that treatment of breast cancer and colorectal cancer cells with a pan-mTOR inhibitor synergistically increases the growth-inhibitory action of the Akt-inhibitor MK2206 which corresponded with changes in the incorporation of radiolabelled choline.

## Materials and methods

### Materials

Breast cancer cells (MDA-MB 468 and SKBr3) and colon cancer cells (HCT8, HCT116 and SW620) were purchased from American Type Cell Culture Collection (ATCC). Unless stated otherwise all chemicals were purchased from Sigma-Aldrich chemical company. The inhibitors used in this study, MK2206 and AZD8055 targeting Akt and mTORC1 and 2 respectively, were obtained from Cambridge Biosciences (Cambridge, UK).

### Cell culture and cytotoxicity assay

Cells were grown in Dulbecco’s Modified Eagle medium supplemented with 10% foetal bovine serum containing penicillin (100 units/ml) / streptomycin (10 μg/ml) in 75cm^2^ tissue culture flasks and incubated at 37 °C in a CO_2_ incubator (Thermo-Scientific UK) with a humidified atmosphere of 95% air; 5% CO_2_. When they reached confluency cells were detached with trypsin and after addition of medium the cells were counted in a haemocytometer (Improved Neubauer, Weber Scientific International UK) and cell suspensions of 30 × 10^3^ cells/ ml prepared. Cell suspension (100 μl) was added to rows 2–10 (columns B to G) and 100ul of medium added to row H (background) of 96 well plate. The plates were then incubated at 37 °C for 24 h to allow cells to adhere. Medium (100 μl) was added to row B and H. To the remaining cells were added either MK2206 (0.04-20 μM) or AZD8055 (8-1000 nM) in increasing concentrations to rows C to G and the plate incubated for a further 72 h. The cytotoxic effect of each drug was assessed by the MTT (3-(4, 5-dimethylthiazol-2-yl)-2, 5- diphenyltetrazolium bromide) assay. Briefly medium was removed from the wells then replaced with medium containing 0.5 mg MTT/ml and the plates incubated in the incubator at 37 °C for 1-2 h depending on the time taken for the cells to appear dark purple. The medium was then removed and the purple aqueous-insoluble crystals that formed in the cells by reduction of MTT were solubilised by addition of 100 μl of DMSO. After a brief (10s) agitation the absorbance was measured in a multiwell platereader with the absorbance filter at 540 nm. The IC_50_ values of each drug were calculated using CompuSyn software (ComboSyn, Paramus, NJ) from the absorbance of drug-treated cells expressed as a percentage of absorbance of control (untreated) in well 2.

To determine the effect of combining MK2206 and AZD8055 on proliferation, cells were set up as for the dose curves but before adding the range of drug doses, cells were treated with a dose of the other drug that was known to appreciably (30–40%) decrease cell proliferation. The dose curves with and without combined treatment were then used to calculate the combination index as described by Chou and Talalay (Chou 2010) [13] using the following formula:

$$ CI= Xa/ Xb+ Ya/ Yb. $$

Suitable drug concentrations (Xa, Ya) are used such that their combined effect reduces cell viability to about 20–60% that of the control. Xb and Yb are the concentrations of each drug which when used alone produce the same level of growth inhibition as the combination. The data obtained from the dose curves in the presence and absence of the second drug were also subject to Isobolomic analysis.

### Western blot analysis of Akt, p-Akt (Ser473), mTOR and p-mTOR (Ser2481) protein expression

MDA-MB 468 cells were treated with the MK2206, AZD8055 or the two drugs combined for 24 h and then washed with 5 ml ice cold PBS wash before harvesting by scraping with 150 μl of lysis buffer (PBS containing 1 mM DTT, a protease inhibitor cocktail, 10 mM sodium pyrophosphate and 1 mM sodium orthovanadate). Samples were kept frozen until they were lysed with a probe sonicator for 30 s. Protein concentration was determined using the bicinchonininic acid (BCA) assay. Lysates (25 μg protein/well) were resolved on 4%–12% acrylamide bis–tris gels (Invitrogen) and the proteins transferred to Immobilon-P polyvinylidene difluoride membranes (Millipore) at 30 V for 1.5 h using a wet transfer system (Invitrogen). Membranes were blocked for 1 h at room temperature in sea block PBS for the Akt antibody and 1% (*w*/*v*) BSA for the p-Akt antibody. Membranes were blotted with antibodies in PBST (PBS containing 0.1% Tween 20) at 4 °C overnight. Akt and p-Akt (1/667 dilution) and mTOR and p-mTOR (1/1000 dilution) were used. Membranes were washed four times in PBST prior to 1 h incubation with the appropriate anti-rabbit fluorescent secondary antibody in PBST in the dark. The secondary antibody used was IRdye680 donkey anti-rabbit secondary antibody (Licor) (1/10000 dilution). Blots were drained and wrapped in cling film to prevent drying and imaged using the Odyssey® CLx LI-CORE imaging system. Images were then analysed using the Odyssey Image Studio Lite (LI-CORE) software.

### Flow cytometry analysis of cell cycle distribution

Cells were seeded in 25cm^2^ flasks with 2 ml medium (1 × 10^6^ cells) and incubated at 37 °C until treated with IC_50_ concentrations of each inhibitor or in combinations for 24 h, then trypsinized. After two PBS washes they were re-suspended in 300 μl PBS followed by fixation with 70% ice-cold ethanol during mixing on a vortex. The fixed cells were kept at −20 °C prior to flow cytometry analysis. For cell cycle analysis, fixed cells were adjusted to 5 × 10^5^ cells/ml and washed 2 times with PBS supplemented with 1% bovine serum albumin (BSA). Then the cells were centrifuged at 1000 g for 5mins and suspended in 1 ml of staining buffer containing 50μg/ml propidium iodide, 50μg/ml ribonuclease A and 0.1% *v*/v triton-x-100 in PBS and incubated for 15 min at room temperature. The stained nuclei were kept at 4 °C and protected from light. Flow cytometry was performed using 488 nm laser light on a FACSCalibur flow cytometer (Becton Dickinson, UK) and CELLQuest software (Becton Dickinson, UK) equipped for fluorescence detection, forward, 90° angle light scatter and doublet discrimination. The data analysis was carried out with Flow-jo cell cycling software.

### [Methyl-^3^H]-choline incorporation

For each experiment 12 flasks (25cm^2^) were seeded with 1 × 10^6^ cells in 2 ml of medium and allowed to attach overnight at 37oC. Cells were then treated by addition of medium (6 flasks) or medium containing AZD8055 (6 flasks) and incubated overnight at 37 °C. To 3 untreated and 3 AZD8055-treated flasks of cells was added MK2206 and the flasks incubated for a further 24 h at 37 °C. Cells were then incubated with fresh medium containing [methyl-^3^H]-choline (37 kBq/ml) for 15 mins [14]. Then the cells were washed 5 times with phosphate buffer saline (PBS) and detached by treatment with trypsin (0.35 ml). After addition of medium (0.35 ml) to neutralise the trypsin half the cell suspension was added into 5 ml of Ultima Gold scintillation fluid (Perkin-Elmer UK) in a scintillation vial (Perkin-Elmer UK) and counted in the liquid scintillation counter (Packard Tricarb2300) for 10 mins per vial. The remaining cells were centrifuged and washed with 1 ml of PBS and the pellet dissolved in 0.1 ml of NaOH (1 M) overnight for determination of protein concentration after neutralization with 0.1 ml of HCl (1 M).

### Protein assay

Protein assay was done by using the BCA protein assay kit (Sigma-Aldrich UK) with Bovine serum albumin (BSA) as a standard according to manufacturer’s protocol.

### Statistical analysis

Statistical differences between means were determined using the data acquired from at least 3 independent experiments. Significance was set at *p* < 0.05. Statistical analysis for finding IC_50_ was performed using CompuSyn software (ComboSyn, Paramus, NJ). Combination index (CI) were calculated from fractions of dose-effect curves by Chou and Talalay [13] for drug interactions.

## Results

**Fig. 1:**
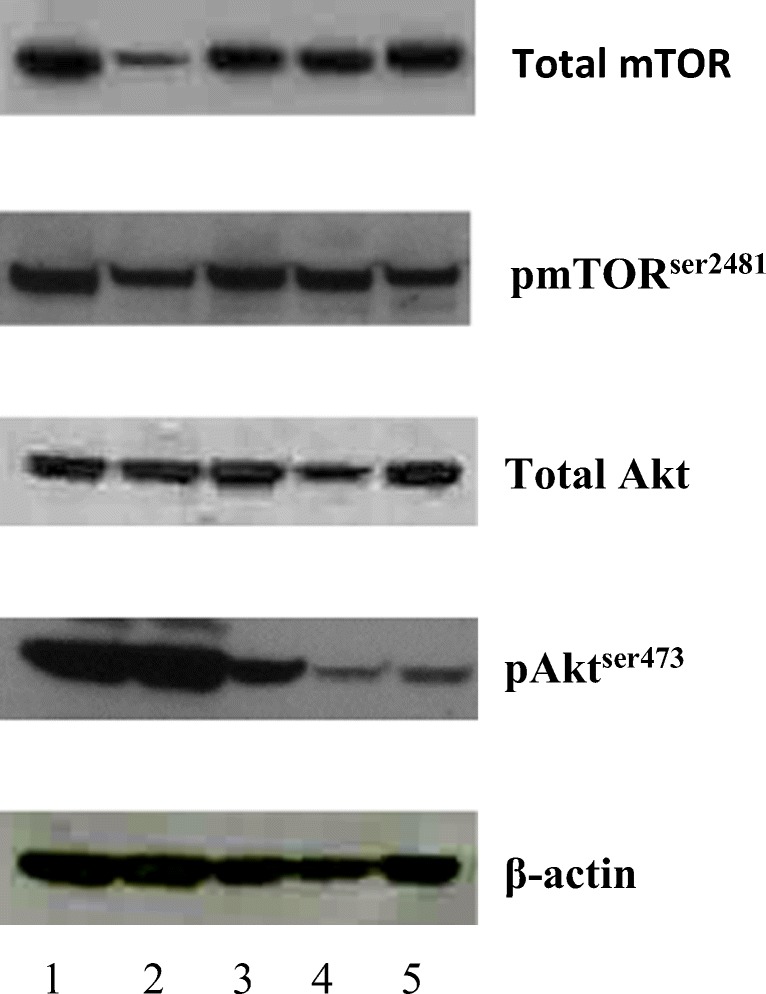
Expression of total mTOR, p-mTOR^ser2481^, total Akt and pAkt^ser473^ in SKBr3 (Lane 1), MDA-MB-468 (Lane 2), HCT8 (Lane 3), HCT116 (Lane 4) and SW620 (Lane 5)

Up-regulation of PI3K/Akt/mTOR is an important indicator for treatment with inhibitors of this pathway therefore an initial screen of phospho-Akt^ser473^ and phosphor-mTOR^ser2481^ expression was carried out in 2 breast and 3 colorectal cancer cell lines. The western blots are shown in Fig. 1. SKBr3, MDA-MB-468 and HCT8 cells expressed p-Akt^ser473^ at high levels. All lines expressed similar levels of p-mTOR^ser2481^ protein.

**Fig. 2:**
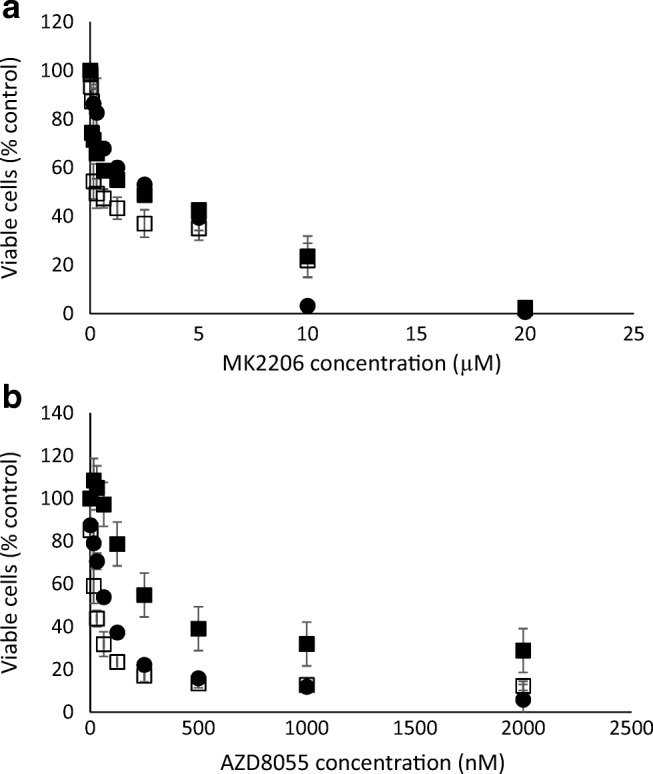
Inhibition of proliferation of SKBr3 (empty squares), MDA-MB-468 (filled circles) and HCT8 (filled squares) cancer cells by treatment with increasing concentrations MK2206 (**a**) and AKD8055 (**b**) determined using the MTT assay. Results expressed as absorbance at 540 nm relative to cells in untreated wells (%)

**Fig. 3:**
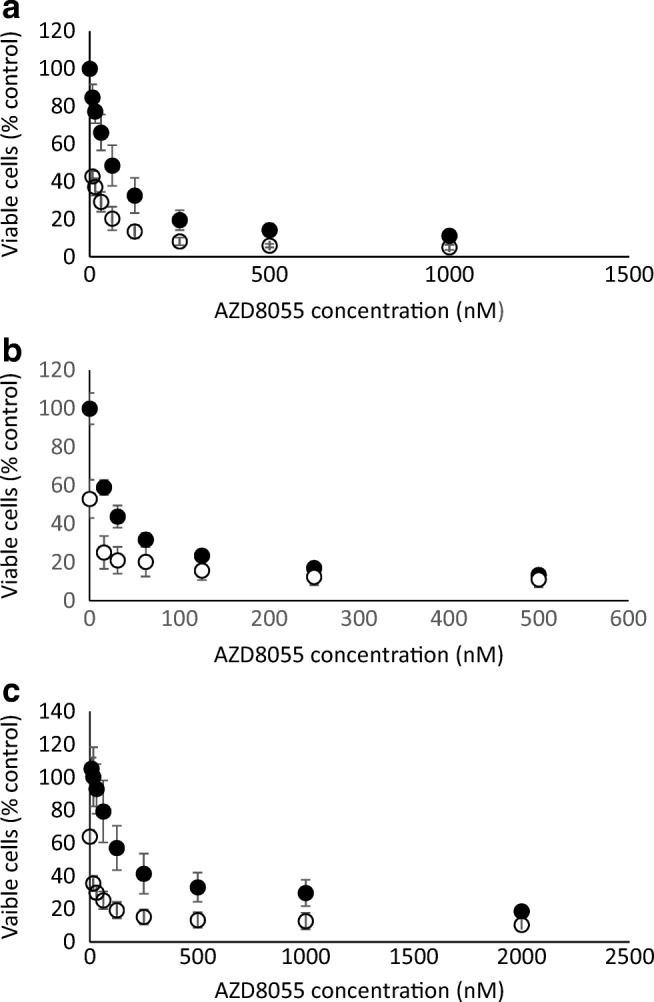
Inhibition of proliferation of SKBr3 (**a**), MDA-MB-468 (**b**) and HCT8 (**c**) cancer cells by treatment with increasing concentrations AZD8055 alone (filled circles) and in the presence of an IC40 dose MK2206 (open circles) determined using the MTT assay. Results expressed as absorbance at 540 nm relative to cells in untreated wells (%)

**Fig. 4:**
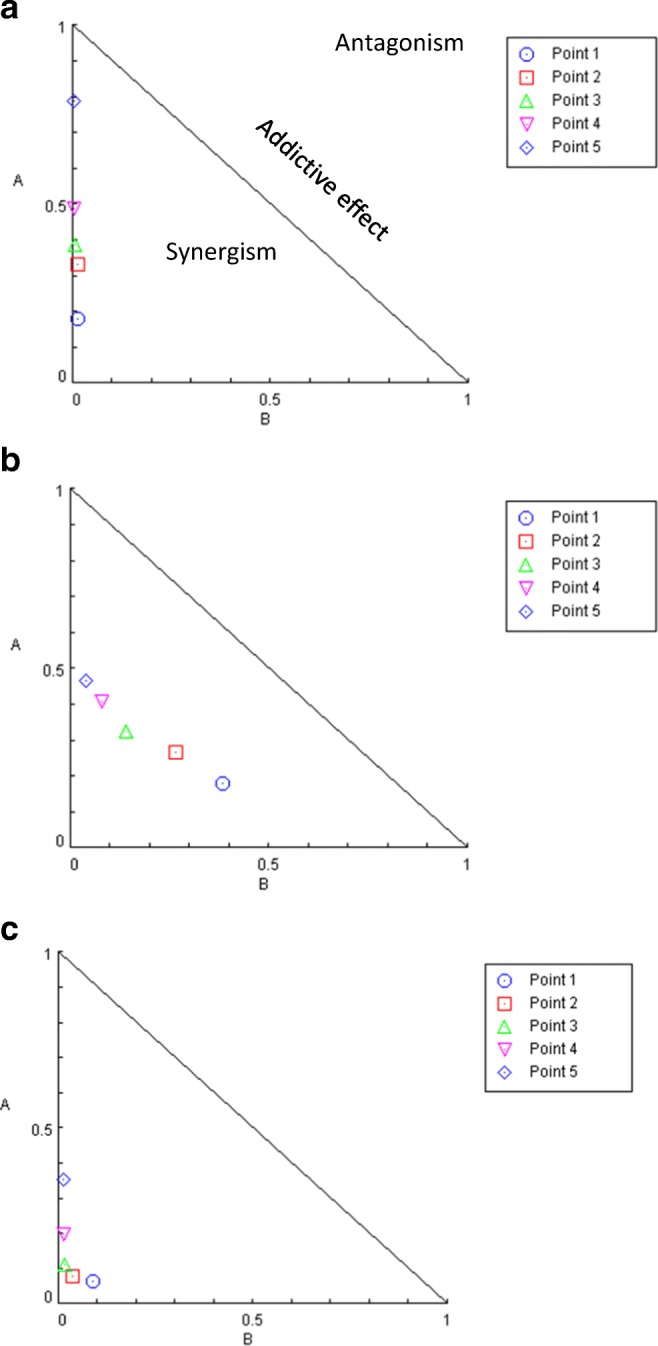
Normalised isobolograms (produced using CompuSyn software (ComboSyn, Paramus, NJ)) for the combination of MK2206 (x-axis) and AZD8055 (y-axis) treatment of SKBr3 (**a**), MDA-MB-468 (**b**) and HCT8 (**c**) cells

Figure 2 shows the effect of the pan-Akt inhibitor MK2206 and the pan-mTOR inhibitor AZD8055 on the growth rate of the breast cancer cell lines MDA-MB-468 and SKBr3 and the colorectal cell line HCT8. The IC_50_ doses of each drug are shown in Table 1. The effect of combining AZD8055 with a clinically relevant dose of MK2206 [3] that decreases cell growth by 30–40% (310 nM) is shown in Fig. 3. The combined effect of these drugs were then assessed using the combination index Table 2) and found to be synergistic. The results were confirmed using isobolomics using CompuSyn and the isobolograms displayed in Fig. 4.

**Table 1:** IC_50_ doses of MK2206 and AKD8055 for breast and colorectal cancer cells treated for 72 h

Cell lines	Signalling inhibitors	IC_50_
MDA-MB468	MK2206	2.16 ± 0.206 μM
	AZD 8055	65.1 ± 11 nM
HCT8	MK2206	1.58 ± 0.66 μM
	AZD8055	181.77 ± 46.05 nM
SKBr3	MK2206	0.479 ± 0.15 μM
	AZD8055	19.407 ± 5.39 nM

**Table 2:** Combination indices for breast and colorectal cancer cells treated with MK2206 and AZD8055

Cell lines	Combination Index (CI)
SKBr3	0.334 ± 0.23
HCT8	0.31 ± 0.03
MDA-MB468	0.456 ± 0.27

**Fig. 5:**
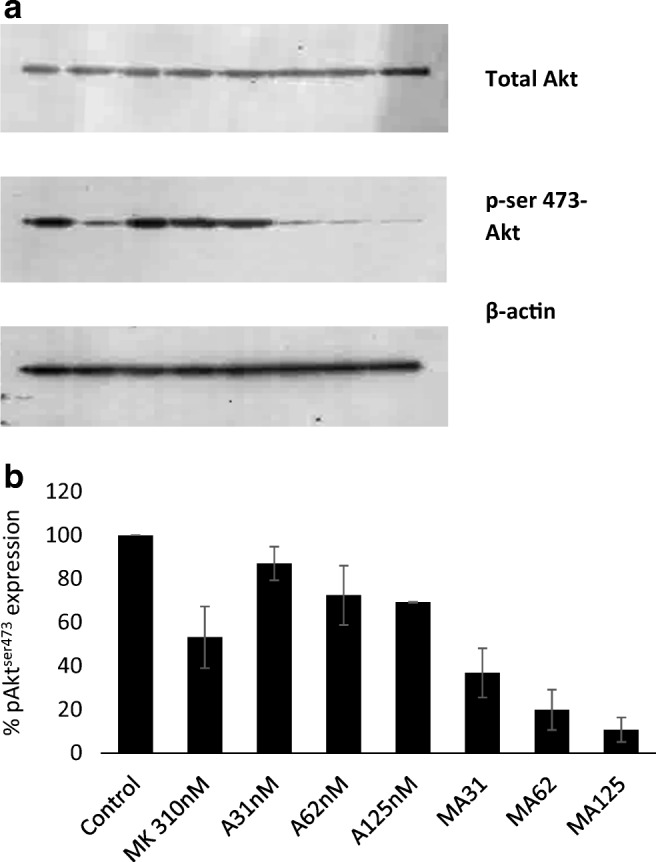
Expression of total and pAkt^ser473^ by MDA-MB-468 cells treated with MK2206, AZD8055 and both combined for 24 h

To confirm that MK2206 and AZD8055 down-regulated pAkt^ser473^ [15, 16] the expression of p-Akt^ser473^ and total Akt by MDA-MB-468 cells untreated and treated with MK2206 (310 nm), AZD8055 (31, 62 and 125 nM) or both drugs combined was determined. The results shown in Fig. 5 demonstrate that both drugs decrease the level of pAkt^ser473^ and that combining MK with AZD resulted in greater decreases in pAkt^ser473^ levels.

**Fig. 6:**
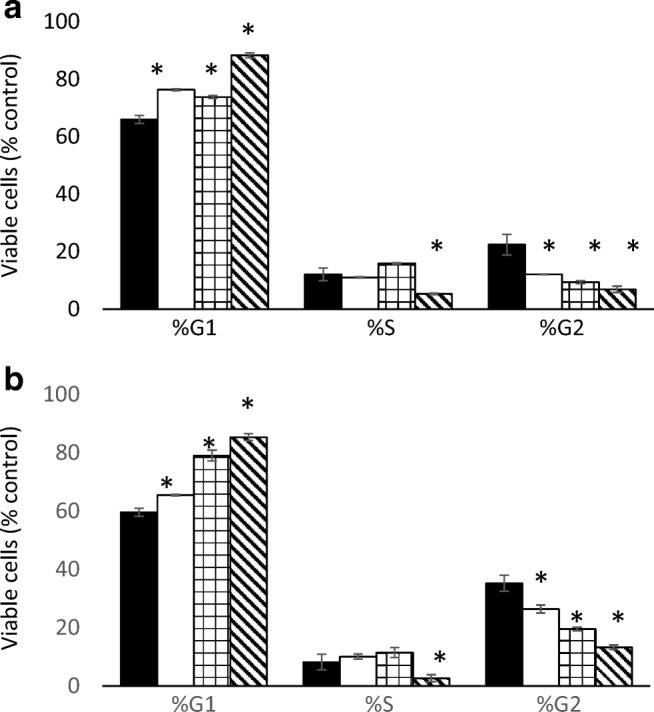
Effect of treatment of SKBr3 (**a**) and MDA-MB-468 (**b**) cells for 24 h with MK2206 (white), AKD8055 (squares) and both combined (diagonal lines) (untreated Black) on cell cycle distribution using flow cytometry analysis (* indicates significant difference (*p* < 0.05) from control)

The effects of MK2206 and AZD8055 alone or in combination on cell cycle distribution was determined in SKBr3 and MDA-MB-468 cells and the results shown in Fig. 6. Each drug alone significantly (*P* < 0.05) increased the proportion of cells in G0/G1 and decreased the proportion in G2 in both cells lines. The two drugs together significantly (P < 0.05) increased the proportion of cells in G0/G1 and decreased the proportion of cells in G2 compared with either drug alone in both cell lines.

**Fig. 7:**
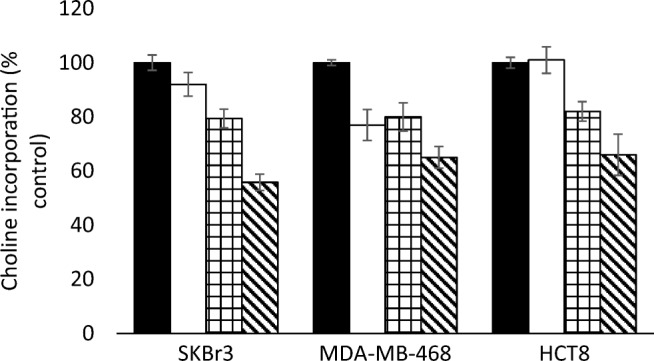
Choline incorporation by SKBr3, MDA-MB-468 and HCT8 cells untreated (black) and treated with MK2206 (white), AKD8055 (squares) and both (diagonal lines) combined and incubated with [methy-^3^H] choline for 15 min. (Units: cpm/mg protein expressed relative to untreated cells (%))

Treatment of SKBr3, MDA-MB-468 and HCT8 cells with AZD8055 and MK2206 significantly (*p* < 0.05) decreased choline incorporation compared with treatment with either MK2206 or AZD8055 alone (results shown in Fig. 7).

## Discussion

Inhibition of intracellular signalling pathways is an attractive strategy for anticancer treatment. However due to feedback mechanisms and cross-talk cancer cells are able to minimise the proliferation-attenuating effects of signal pathway inhibiting drugs when administered as single agents. The PI3K/Akt/mTOR pathway is up-regulated in cancer due to several mechanism including activating mutations of PI3K and Akt, inactivating mutations of PTEN and over-expression of upstream receptors such as the tyrosine kinase human epithelial receptor family (HER). An initial screen of several cell lines confirmed high phospho-Akt^ser473^ expression in SKBr3, MDA-MB-468 and to a lesser extent in HCT8 cells.

SKBr3 cells over-express HER-2 [10, 17] but are wild type for PI3K and PTEN, MDA-MB-468 cells harbour mutant PTEN [17] and HCT8 are mutant for PIK3CA [18]. MK2206 and AZD8055 synergistically inhibited proliferation of SKBr3, MDA-MB-468 and HCT8 cells. This would suggest that the underlying mechanism of PI3K/Akt/mTOR up-regulation is not necessarily a factor in the synergistic growth inhibitory effect of pan-Akt and pan-mTOR inhibitors.

Treatment of cancer cells with AZD8055 [19, 20] and MK2206 [21, 22] has been shown to cause accumulation of cells in G0/G1. Thus English et al. [19] found that response to treatment of uterine serous carcinoma cell lines, especially cells over-expressing HER2, with 1 μM or 5 μM AZD8055 was associated with G0/G1 cell cycle arrest in a concentration-dependent manner. Qian et al. 2016 [20] has reported that the pan-mTOR kinase inhibitors MTI-31 and AZD8055 induce G0/G1 cell cycle arrest corresponding with suppression of cyclin D1 and c-Myc in HER2+/PIK3CA mutant breast cancer cells. Similarly for the pan-Akt inhibitor, MK2206, studies [21, 22] have demonstrated that treatment with this drug induced G0/G1-phase arrest which was associated with a marked decrease in cyclin D1 levels in hepatocellular carcinoma HepG2 cells. In agreement with these studies we have found MK2206 and AZD8055 induced G0/G1 cell cycle arrest in breast cancer and colorectal cancer lines whilst the combination of MK2206 with AZD8055 exacerbated G0/G1 cell cycle arrest.

Progression of human mammary epithelial cells to a malignant phenotype is associated with altered membrane choline metabolism [23] due to changes in the expression of enzymes that control anabolic and catabolic reactions of choline metabolism resulting in increased levels of choline-containing precursors and breakdown products of membrane phospholipids [24]. Increased phosphocholine levels are frequently found in cancer tissue [25, 26] and studies have shown that the uptake and phosphorylation of radiolabelled choline by cancer cells correlates with proliferation rate [11]. Here choline uptake was decreased by breast cancer and colorectal cancer cells when treated with both MK2206 and AZD8055 but MK2206 alone only decreased choline uptake significantly in the breast cancer cell lines. Studies using ^31^P-NMR spectroscopy have demonstrated that inhibition of PI3K/AKT/mTOR signalling pathway by PI-103 reduced choline kinase activity, resulting in decreased in PCho in colon cancer cells [27]. Venkatesh et al. [28] has also demonstrated that treatment with the PI3K inhibitor LY294002 and mTOR inhibitor everolimus decreased phosphocholine levels associated with decreased in choline kinase activity in glioblastoma cell lines. However other studies have shown that phosphocholine content of cancer cells isn’t consistently altered by treatment with PI3K/Akt/mTOR inhibitors [29].

In summary treatment of tumour cells with pan-Akt and pan-mTOR synergistically inhibited the growth of cancer cells and was associated with arrest in G0/G1. Radiolabelled choline uptake was most strongly decreased by dual drug treatment and in combination with PET may be a useful non-invasive indicator of efficacious drug combinations.
